# Ethnic and residential differences in adherence to the 24-h movement guidelines and self-reported physical fitness among Zhuang and Han adolescents in southern China

**DOI:** 10.3389/fpubh.2025.1631277

**Published:** 2025-12-04

**Authors:** Hongsheng Qian, Jingwang Tan, Jiaxing Tang, Yu Zou

**Affiliations:** 1 School of Physical Education, Wuhan Sports University, Wuhan, China; 2 Department of Sport and Exercise Science, College of Education, Zhejiang University, Hangzhou, China; 3 School of Physical Education, Xiangnan University, Chenzhou, China

**Keywords:** physical fitness, physical activity, screen time, sleep duration, movement guidelines, ethnicity, residence

## Abstract

**Background:**

The 24-h movement guidelines integrate physical activity (PA), screen time, and sleep for health benefits, yet little is known about how adherence to these guidelines relates to physical fitness (PF) among adolescents, particularly in the context of ethnic and residential differences. This study examines the independent and combined associations of adhering to the 24-h movement guidelines with self-reported PF among Han and Zhuang adolescents in southern China, while exploring potential differences by residence and ethnicity.

**Methods:**

Cross-sectional data from 6,913 adolescents were analyzed using a validated self-reported questionnaire to assess adherence to the 24-h movement guidelines and PF components (cardiorespiratory fitness, cardiorespiratory fitness, speed/agility, muscular strength, and flexibility). Generalized linear models were used to examine associations.

**Results:**

In this sample, only 1.6% of participants met the overall guidelines. Meeting more guidelines was significantly (*p* < 0.001) associated with better self-reported PF indicators. The combinations containing moderate-to-vigorous physical activity (MVPA) guideline were significantly (*p* < 0.05) associated with self-reported PF indicators enhancement compared to those combinations without MVPA guideline. Meeting screen time guideline was significantly (*p* < 0.05) associated with more self-reported PF indicators, but not in the case of meeting sleep duration guideline. Most of the results were similar for Han and Zhuang ethnicity, whereas residence differences exerted a significant impact on the relationship between adherence to “Screen only,” “Sleep only,” and “Screen + sleep” guideline and self-reported PF indicators.

**Conclusion:**

Our findings highlight the critical role of adhering to the 24-h movement guidelines, mainly MVPA, in improving self-reported PF among adolescents and underscore the need to address urban–rural disparities in promoting the 24-h movement guidelines.

## 1 Introduction

Physical fitness (PF) is defined as the capacity to perform physical activity (PA) and encompasses qualities such as cardiorespiratory fitness (also called aerobic capacity), muscular fitness, speed/agility, and flexibility (1). PF is widely acknowledged as a vital marker of physical and mental well-being in children and adolescents (2, 3). Extensive evidence has demonstrated positive associations between PF and various health indicators, such as cardiovascular and metabolic health (4, 5), motor competence (6), bone health (7), mental well-being (8), and a lower risk of non-communicable diseases (9, 10). Additionally, PF is linked to cognitive function and academic performance in children (11, 12). Unfortunately, declines in cardiorespiratory fitness and strength have been observed in recent decades (13–15), likely driven in part by rising sedentary behavior, insufficient PA, and environmental constraints on active living (16–20). This trend underscores the urgency of promoting PF as a public health priority.

In recent years, growing evidence has shown independent associations between sufficient PA (16, 17), limited screen time (18, 19), and appropriate sleep duration (19, 20) with higher levels PF in children and adolescents. However, previous research has also reported contrary findings (21, 22). These results highlight the need for holistic approaches to enhance PF. In 2016, Canada released the world’s first 24-h movement guidelines for children and adolescents, recommending ≥ 60 min of moderate-to-vigorous physical activity (MVPA) daily, ≤ 2 h of screen time, and age-appropriate sleep duration (9–11 h for children aged 5–13 and 8–10 h for adolescents aged 14–17) (23). This paradigm shift emphasizes integrated movement behaviors for health (24) and has been widely adopted by scholars in numerous countries (24–26), including China (27).

To our knowledge, only five empirical studies have explored the association between adherence to the 24-h movement guidelines and PF in children and adolescents. The studies conducted by Carson et al. (28) in Canada, Chen et al. (29) and Cai et al. (30) in China, as well as Tapia-Serrano et al. (31) in Spain, all found that meeting the three guidelines was associated with better PF or some components of PF. Conversely, the study conducted by Tanaka et al. in Japan reported that meeting all three guidelines was unrelated to PF (i.e., muscular fitness, cardiorespiratory fitness, and flexibility) (32). In addition, these studies also revealed significant disparities in the prevalence of meeting the guidelines (0.9% or 12.4% in China, 5.7% in Spain, 9.1% in Japan, and 17% in Canada) (28–32), as well as variability in the relationship between specific guideline combinations and PF indicators (28, 29, 32). These discrepancies may stem from study design heterogeneity, as well as geographical and demographic factors such as country, residence, and ethnicity. Geographical disparities imply varying influences of institutional norms, socioeconomic and cultural contexts, built environments, lifestyles, and community dynamics on human behavioral patterns and health outcomes (33, 34). Ethnic factors also play a critical role in health research, as various health outcomes are shaped by genetic inheritance and ethnicity-specific characteristics (e.g., dietary habits, living environment, traditions and lifestyles) (35–37). Given these influences, one study has called for further investigation into how geographical context and ethnicity may moderate the association between adherence to the 24-h movement guidelines and health indicators (26). However, no study has examined geographical or ethnic differences in the association between the 24-h movement guidelines and PF among adolescents, highlighting the need for further research in diverse regions and ethnic groups.

China is a multi-ethnic country in which the Han majority comprises 91% of the population. The Zhuang, the largest ethnic minority, number around 20 million (38) and is concentrated in the Guangxi Zhuang Autonomous Region (Guangxi) of southern China. Guangxi is a subtropical, high-precipitation border province noted for its karst landscapes, cultural diversity, and comparatively low economic development (39). Adherence to health-related lifestyle behaviors—including smoking abstinence, limited alcohol consumption, PA, and a balanced diet—remains low across Guangxi, with notable urban–rural disparities (40). Compared with Han adolescents, Zhuang adolescents exhibit distinct dietary patterns (e.g., frequent consumption of wild edible plants and corn-based staples) (35, 41, 42) and actively participate in ethnic-specific cultural activities, including traditional festivals, folk singing, and group dancing (43). These characteristics suggest that Han and Zhuang adolescents may display distinct 24-h movement behaviors and PF. However, the association between adherence to 24-h movement guidelines with PF and its components among Chinese Han and Zhuang adolescents in southern China remains to be well established, and it remains unknown whether the relationship differs by ethnicity or residence. Addressing this gap, this study aims to examine the associations between adherence to the 24-h movement guidelines and self-reported PF among Han and Zhuang adolescents in southern China, while exploring potential differences by residence and ethnicity. The findings are intended to inform targeted interventions to improve PF in diverse adolescent sub-populations.

## 2 Materials and methods

### 2.1 Participants and procedure

This study, supported by the local education commission of Guangxi Zhuang Autonomous Region, was conducted between July and August 2024 in 12 public primary schools across two cities with a high concentration of the Zhuang ethnic group. Previous research indicated that children aged 10 years or older possess adequate reading comprehension skills to complete self-report questionnaires (44). In China, fourth-grade students are typically around 10 years old. Therefore, students in the fourth grade and above were included as qualified participants.

The aim of the study was presented to the participants and their guardians in a written format. This survey is intended to collect data about demographic information, self-reported PA, screen time, sleep duration and PF. Participants voluntarily completed the questionnaire using electronic devices (e.g., computers or mobile phones). Students without personal access to electronic devices were encouraged to use their parents’ equipment. If electronic devices were unavailable, students completed a paper-based questionnaire, and school staff manually converted into an electronic format. Only participants who provided consent were able to proceed to the formal data collection pages. All data were collected and analyzed anonymously. The study received ethical approval from Wuhan Sports University (No. 2025019).

**Figure 1:**
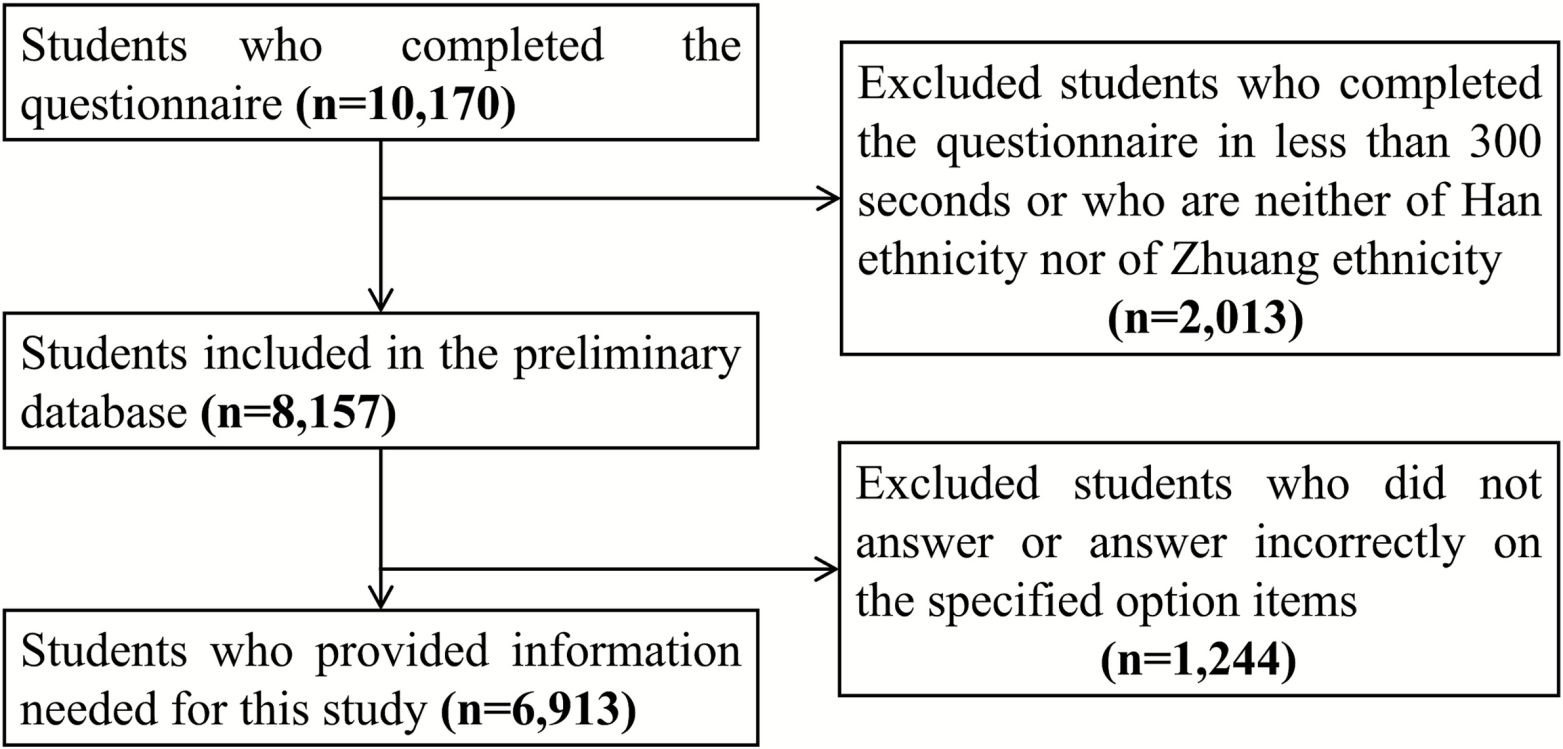
Detailed process used for cleaning invalid and missing data.

A total of 10,170 participants completed the questionnaire. We excluded those who (i) completed the questionnaire in < 300 s, (ii) were neither Han nor Zhuang, or (iii) had implausible values for age, physical activity, screen time, or sleep. After considering the availability of the necessary variables, the final sample for analysis comprised 6,913 students, as shown in Figure 1.

### 2.2 Measures

#### 2.2.1 Demographics

Demographic information was gathered as the initial section of the questionnaire, encompassing age, sex, grade, number of siblings, household-registered residence (Rural: residents registered in a village, suburban: residents registered in a township or in the peri-urban districts of a city, and urban: residents registered within the city proper of a county-level city or above), and household-registered ethnicity. These variables have been accounted for in statistical analyses to mitigate confounding bias (29, 31, 44–47). Thus, during subsequent statistical analysis, these variables (excluding those being examined for interaction effects) were regarded as covariates.

#### 2.2.2 24-h movement behaviors

PA was measured using a reliable and valid item sourced from the Health Behavior in School-Aged Children (HBSC) survey (48). The item was formulated as follows: “How many days did you engage in moderate-to-vigorous physical activity (MVPA) for at least 60 min in the past week? (0 = none, 1 = 1 day, 2 = 2 days, 3 = 3 days, 4 = 4 days, 5 = 5 days, 6 = 6 days, 7 = 7 days).” MVPA was defined as activities that induced at least moderate breathlessness and sweating during daily life, physical education classes, and other exercise sessions (49).

Screen time was assessed using the relevant items in the HBSC survey (48). The time spent on three distinct screen-based behaviors (i.e., TV/movies, video games, and other screen-based activities) over the past 7 days were measured. The average daily screen time was computed by considering school days and weekend days. For example, the formula for calculating the average daily TV/movie time was [(daily TV/movies time on school days × 5) + (daily TV/movies time on weekend days × 2)]/7.

Sleep duration was measured using the entry on sleep duration from the Pittsburgh Sleep Quality Index (PSQI): “How many hours did you actually sleep at night in the past month?” The Chinese version of the PSQI has been validated among Chinese adolescents (50).

Furthermore, all the above-mentioned items have been widely applied in research involving Chinese children and adolescents, including Zhuang and other ethnic minorities (27, 29, 46, 51)—confirming their suitability for the target population of the present study. According to the Canadian 24-h Movement Guidelines for Children and Youth, reporting ≥60 min of MVPA daily was considered to meet the PA guideline; ≤2 h per day of screen time was considered to meet the screen time guideline; 9–11 h of sleep duration for children aged 5–13, and 8–10 h of sleep duration for adolescents aged 14–17, respectively, were considered to meet the sleep guideline (23).

#### 2.2.3 Physical fitness

PF was evaluated using the Chinese-version of the International Fitness Scale (IFIS-C). This scale has been proven to be a reliable tool for assessing PF (Cronbach’s *α* = 0.72) (52, 53) and widely applied in Chinese children and adolescents (29, 51). For the sample of the present study, the test–retest reliability remained satisfactory (Cronbach’s α = 0.832). The scale consists of five components: general physical fitness, cardiorespiratory fitness, speed and agility, muscular strength, and flexibility. Responses for each component are measured on a 5-point Likert scale, with options ranging from “very poor” to “very good.”

### 2.3 Analytical methods and tools

All statistical analyses were carried out with the SPSS software (version 26, IBM Corporation, Armonk, NY, USA). Descriptive statistical methods were employed to present the basic characteristics of the participants. For continuous variables (e.g., age), data were reported as mean ± standard deviation (SD), while categorical variables (e.g., ethnicity and residence) were presented as counts and percentages.

Two sets of pairwise regression models were constructed. In the first set, all possible pairwise comparisons were conducted across levels of the number of 24-h movement guidelines met (0, 1, 2, or 3), resulting in six independent models (e.g., 3 vs. 2, 3 vs. 1, 3 vs. 0, etc.), with *p*-values adjusted using the Šidák method. In the second set, we examined all possible pairwise contrasts among the eight possible combinations of guideline adherence (e.g., all vs. sleep only, MVPA only vs. None, etc.), yielding 28 additional independent models. Given the large number of comparisons, the Holm–Bonferroni method was applied to balance rigorous error control with statistical power. To explore whether the association between guideline adherence and PF differed by ethnicity or residence, interaction terms were included in the primary models. Generalized linear models (GLMs) with normal distribution and identity link function were employed to estimate the aforementioned relationships (54–56). The unstandardized regression coefficient (B) represents the adjusted mean difference in PF scores between the compared groups, and statistical significance was defined as *p* < 0.05 after adjustment for multiple comparisons. Given the observed SD of PF scores (SD = 0.81, as shown in Supplementary Table S1), a *B* value of ≥ 0.405 (0.5 SD) was considered a moderate, potentially meaningful change, and ≥ 0.162 (0.2 SD) a small but detectable change, based on established benchmarks for patient-reported outcomes (57, 58).

## 3 Results

### 3.1 Descriptive characteristics of the participants

The descriptive characteristics of the participants, classified by residence and ethnicity, are presented in Table 1. The sample consisted of 21.5% Han ethnicity (45.1% girls) and 78.5% Zhuang ethnicity (49.9% girls), with a mean age recorded of 11.6 years (SD = 1.0), and 63.0, 18.0, and 19.0% of participants lived in rural, suburban and urban regions, respectively. Regarding the exposure variables, 28.0% of the participants did not meet any guideline, while only 1.6% met all three guidelines. 2.4, 10.1, and 37.9% of participants met only the MVPA guideline, the screen time guideline, and the sleep guideline, respectively. A greater proportion of Han participants met all three guidelines, but a lower proportion met guideline for sleep duration. A greater proportion of rural participants met guideline for sleep duration, but a lower proportion met guideline for MVPA (see Supplementary Table S2 for details). Regarding the outcome variables, approximately 50% of the participants perceived themselves as “average.” A higher proportion of participants reported evaluations of “good” and “very good” compared to those reporting “poor” and “very poor.” Moreover, a greater proportion of urban participants reported evaluations of “very good” (as shown in Supplementary Table S2).

**Table 1:** The descriptive characteristics of the participants classified by residence and ethnicity, respectively (*n* = 6,913).

Variables		Total	Ethnicity		Residence		
			Han	Zhuang	Rural	Suburban	Urban
Age (years old)		11.6 ± 1.0	11.6 ± 1.0	11.6 ± 1.0	11.6 ± 1.0	11.6 ± 1.0	11.6 ± 1.0
Gender	Male	3,537 (51.2)	817 (54.9)	2,720 (50.1)	2,210 (50.7)	650 (52.3)	677 (51.6)
	Female	3,376 (48.8)	670 (45.1)	2,706 (49.9)	2,146 (49.3)	594 (47.7)	636 (48.4)
Siblings	Yes	1,136 (16.4)	241 (16.2)	895 (16.5)	582 (13.4)	238 (19.1)	316 (24.1)
	No	5,777 (83.6)	1,246 (83.8)	4,531 (83.5)	3,774 (86.6)	1,006 (80.9)	997 (75.9)
Ethnicity	Han	1,487 (21.5)	1,487 (100.0)	0	862 (19.8)	297 (23.9)	328 (25.0)
	Zhuang	5,426 (78.5)	0	5,426 (100.0)	3,494 (80.2)	947 (76.1)	985 (75.0)
Residence	Rural	4,356 (63.0)	862 (58.0)	3,494 (64.4)	4,356 (100.0)	0	0
	Suburban	1,244 (18.0)	297 (20.0)	947 (17.5)	0	1,244 (100.0)	0
	Urban	1,313 (19.0)	328 (22.1)	985 (18.2)	0	0	1,313 (100.0)
Grade	4	2,443 (35.3)	530 (35.6)	1913 (35.3)	1,555 (35.7)	428 (34.4)	460 (35)
	5	2,223 (32.2)	460 (30.9)	1763 (32.5)	1,432 (32.9)	373 (30.0)	418 (31.8)
	6	2,247 (32.5)	497 (33.4)	1750 (32.3)	1,369 (31.4)	443 (35.6)	435 (33.1)
Adherence to the 24-h movement guidelines	None	1937 (28.0)	411 (27.6)	1,526 (28.1)	1,187 (27.2)	362 (29.1)	388 (29.6)
	One	3,482 (50.4)	738 (49.6)	2,744 (50.6)	2,276 (52.2)	596 (47.9)	610 (46.5)
	Two	1,381 (20.0)	301 (20.2)	1,080 (19.9)	842 (19.3)	258 (20.7)	281 (21.4)
	Three	113 (1.6)	37 (2.5)	76 (1.4)	51 (1.2)	28 (2.3)	34 (2.6)
	MVPA only	169 (2.4)	44 (3.0)	125 (2.3)	66 (1.5)	49 (3.9)	54 (4.1)
	Screen only	695 (10.1)	166 (11.2)	529 (9.7)	442 (10.1)	106 (8.5)	147 (11.2)
	Sleep only	2,618 (37.9)	528 (35.5)	2090 (38.5)	1768 (40.6)	441 (35.5)	409 (31.2)
	MVPA + screen	62 (0.9)	9 (0.6)	53 (1.0)	27 (0.6)	15 (1.2)	20 (1.5)
	MVPA + sleep	305 (4.4)	52 (3.5)	253 (4.7)	153 (3.5)	59 (4.7)	93 (7.1)
	Screen + sleep	1,014 (14.7)	240 (16.1)	774 (14.3)	662 (15.2)	184 (14.8)	168 (12.8)
General physical fitness	Very poor	93 (1.3)	28 (1.9)	65 (1.2)	61 (1.4)	18 (1.4)	14 (1.1)
	Poor	214 (3.1)	41 (2.8)	173 (3.2)	128 (2.9)	42 (3.4)	44 (3.4)
	Average	3,464 (50.1)	737 (49.6)	2,727 (50.3)	2,223 (51)	612 (49.2)	629 (47.9)
	Good	2,235 (32.3)	485 (32.6)	1750 (32.3)	1,399 (32.1)	428 (34.4)	408 (31.1)
	Very good	907 (13.1)	196 (13.2)	711 (13.1)	545 (12.5)	144 (11.6)	218 (16.6)
Cardiorespiratory fitness	Very poor	93 (1.3)	23 (1.5)	70 (1.3)	68 (1.6)	12 (1.0)	13 (1.0)
	Poor	369 (5.3)	77 (5.2)	292 (5.4)	217 (5)	77 (6.2)	75 (5.7)
	Average	3,513 (50.8)	741 (49.8)	2,772 (51.1)	2,263 (52)	632 (50.8)	618 (47.1)
	Good	2,162 (31.3)	483 (32.5)	1,679 (30.9)	1,345 (30.9)	373 (30)	444 (33.8)
	Very good	776 (11.2)	163 (11.0)	613 (11.3)	463 (10.6)	150 (12.1)	163 (12.4)
Muscular strength	Very poor	112 (1.6)	29 (2.0)	83 (1.5)	75 (1.7)	17 (1.4)	20 (1.5)
	Poor	500 (7.2)	103 (6.9)	397 (7.3)	310 (7.1)	109 (8.8)	81 (6.2)
	Average	3,898 (56.4)	810 (54.5)	3,088 (56.9)	2,521 (57.9)	672 (54)	705 (53.7)
	Good	1887 (27.3)	437 (29.4)	1,450 (26.7)	1,148 (26.4)	359 (28.9)	380 (28.9)
	Very good	516 (7.5)	108 (7.3)	408 (7.5)	302 (6.9)	87 (7.0)	127 (9.7)
Speed/agility	Very poor	105 (1.5)	25 (1.7)	80 (1.5)	72 (1.7)	13 (1.0)	20 (1.5)
	Poor	541 (7.8)	103 (6.9)	438 (8.1)	362 (8.3)	90 (7.2)	89 (6.8)
	Average	3,565 (51.6)	781 (52.5)	2,784 (51.3)	2,280 (52.3)	661 (53.1)	624 (47.5)
	Good	1979 (28.6)	437 (29.4)	1,542 (28.4)	1,250 (28.7)	335 (26.9)	394 (30.0)
	Very good	723 (10.5)	141 (9.5)	582 (10.7)	392 (9)	145 (11.7)	186 (14.2)
Flexibility	Very poor	161 (2.3)	33 (2.2)	128 (2.4)	98 (2.2)	30 (2.4)	33 (2.5)
	Poor	714 (10.3)	163 (11.0)	551 (10.2)	457 (10.5)	131 (10.5)	126 (9.6)
	Average	3,977 (57.5)	834 (56.1)	3,143 (57.9)	2,565 (58.9)	712 (57.2)	700 (53.3)
	Good	1,572 (22.7)	351 (23.6)	1,221 (22.5)	949 (21.8)	289 (23.2)	334 (25.4)
	Very good	489 (7.1)	106 (7.1)	383 (7.1)	287 (6.6)	82 (6.6)	120 (9.1)

Values are mean ± SD or *n* (%); *n* represents unweighted sample counts, and % is weighted sample sizes.

### 3.2 Associations between adherence to the 24-h movement guidelines and self-reported PF indicators

**Figure 2:**
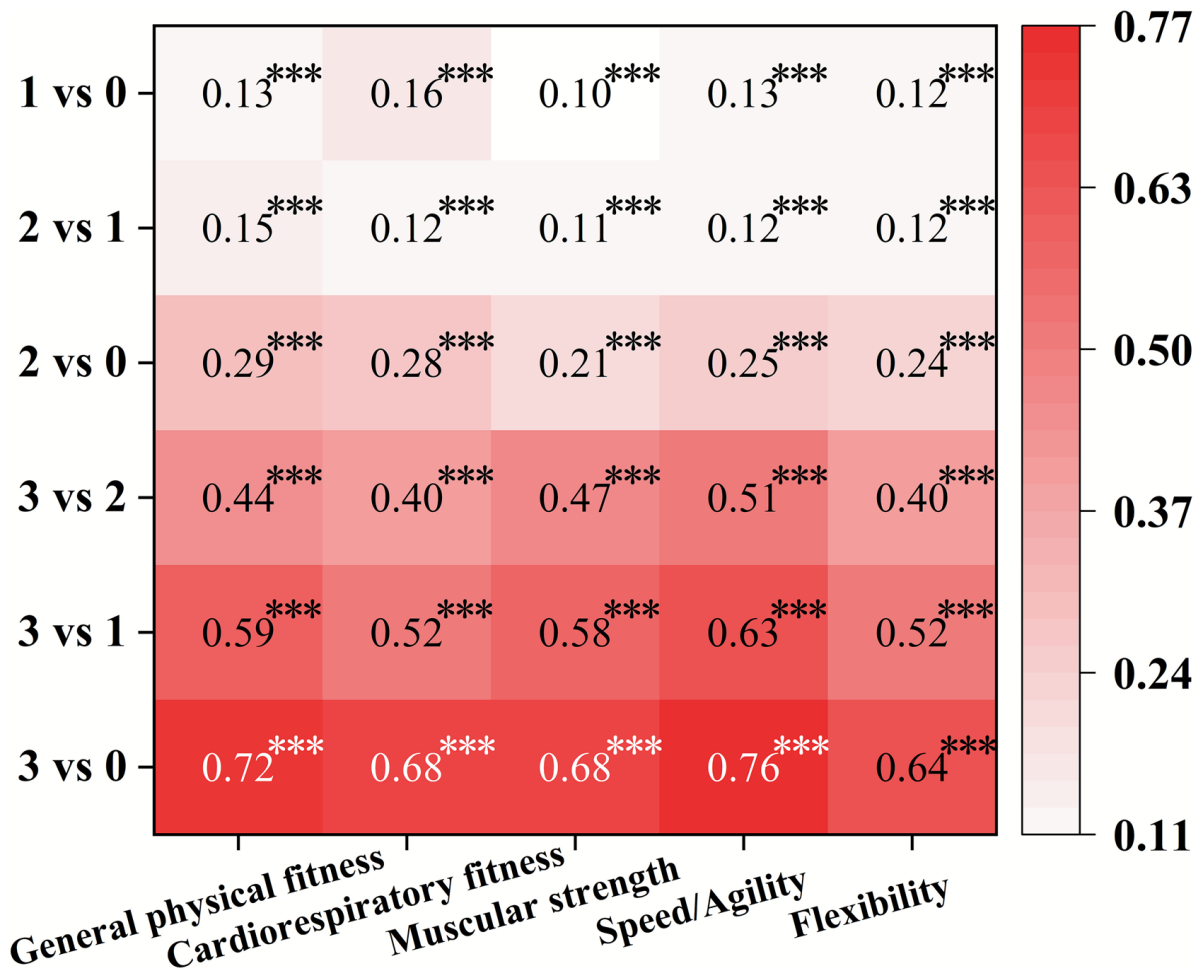
The heatmap visually represents the associations between the number of 24-h movement guidelines met and self-reported PF indicators. The *y*-axis displays pairwise comparisons of guideline adherence levels (e.g., “3 vs. 0” compares meeting all three guidelines versus meeting none), while the *x*-axis lists the five PF components. Each cell shows the unstandardized regression coefficient (*B* value), with statistical significance indicated by asterisks (“***” denotes *p* < 0.001). The map is color-coded according to the magnitude of the *B* value, with darker shades representing higher absolute values, indicating stronger associations.

Figure 2 presents the pairwise comparison of the associations between the number of guidelines met and self-reported PF indicators. Meeting more guidelines was significantly (*p* < 0.001) associated with better PF indicators. Additionally, a consistent dose-effect pattern was observed, with more guidelines met corresponding to higher *B* values.

**Figure 3:**
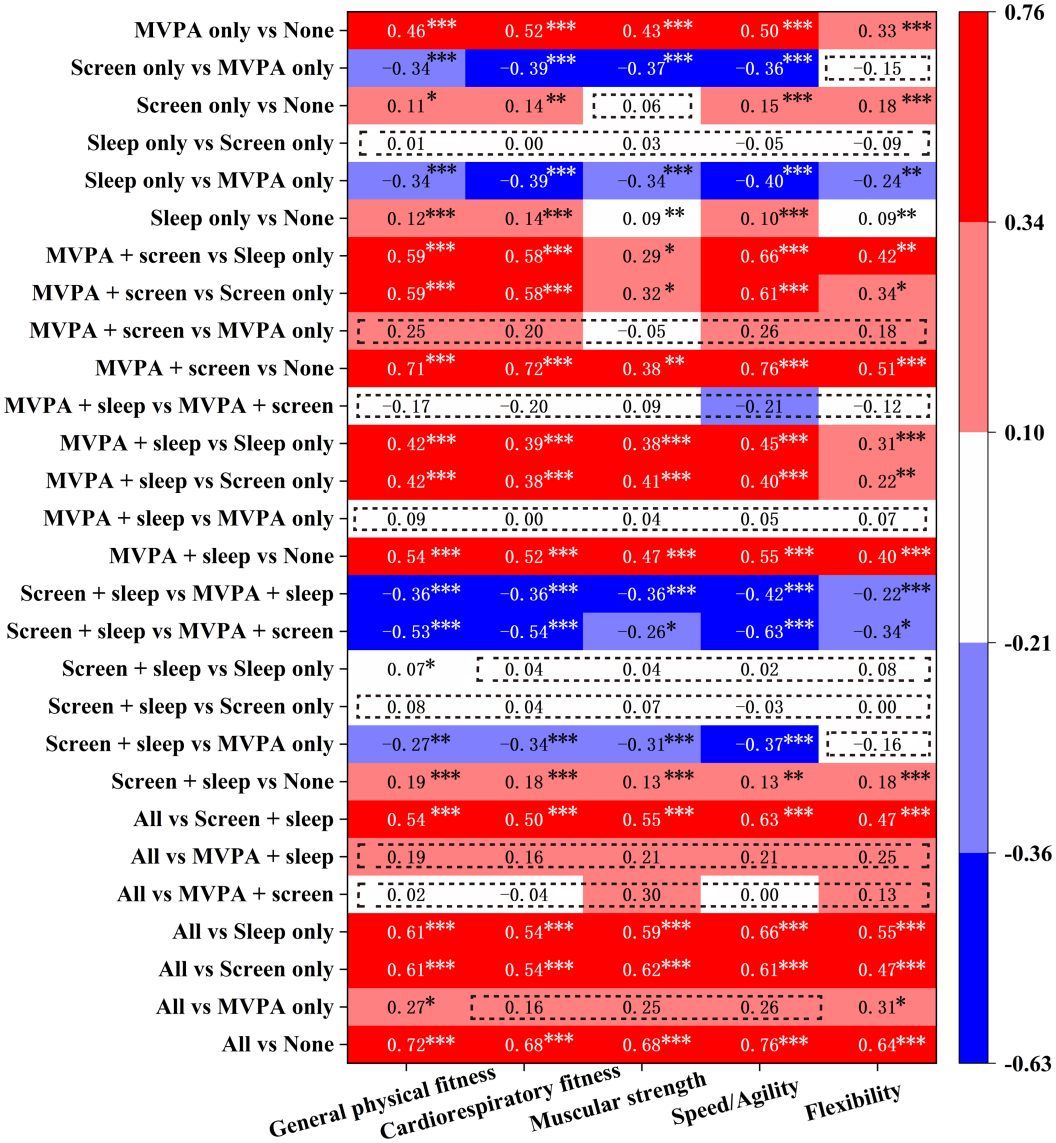
The heatmap visually represents the associations between specific combinations of 24-h movement guidelines met and self-reported PF indicators. The *y*-axis displays pairwise comparisons based on the number of guidelines adhered to (e.g., “All vs. None” compares meeting all three guidelines versus meeting none). The *x*-axis lists the five PF components. Each cell shows the unstandardized regression coefficient (*B* value) for the corresponding association, with statistical significance denoted by asterisks. “***” indicates *p* < 0.001, “**” indicates *p* < 0.01, and “*” indicates *p* < 0.05. Cells with non-significant *B* values (*p* ≥ 0.05) are outlined with a red dashed border. Color intensity is scaled from blue to red according to the *B* values. Dark blue represents the most negative (unfavorable) association, while dark red represents the most positive (favorable) association.

Figure 3 presents the pairwise comparison of the associations between the specific combinations of guidelines met and self-reported PF indicators. Compared to meeting none of the guidelines, any combination of guidelines met was significantly (*p* < 0.05) associated with improved PF indicators except for muscular strength in terms of “Screen only vs. None.” In the pairwise comparison of “Sleep only vs. Screen only,” “MVPA + screen vs. MVPA only,” “MVPA + sleep vs. MVPA + screen,” “MVPA + sleep vs. MVPA only,” “Screen + sleep vs. Screen only,” “All vs. MVPA + sleep,” and “All vs. MVPA + screen,” no significant (*p* > 0.05) enhancement was found with any PF indicators. In addition, the combinations containing MVPA guideline were significantly (*B* > 0.22, *p* < 0.05) associated with the majority of PF indicators enhancement compared to those combinations without MVPA guideline. Finally, by comparing the pairwise combinations of “Sleep only VS Screen only,” “MVPA + screen vs. MVPA only,” “MVPA + sleep vs. MVPA only,” “MVPA + sleep vs. MVPA + screen,” “Screen + sleep vs. Screen only,” “Screen + sleep vs. Sleep only,” “All vs. MVPA + screen,” “All vs. MVPA + sleep,” and “All vs. MVPA + screen,” it is found that meeting the screen guideline was significantly (*p* < 0.05) associated with more PF indicators.

### 3.3 Associations between adherence to the 24-h movement guidelines and self-reported PF indicators by ethnicity and residence

Table 2 presents the associations between the number of guidelines met and self-reported PF indicators, compared to meeting none of the guidelines, stratified by ethnicity and residence. The data show that meeting any number of guidelines was significantly associated with enhanced PF indicators in both Han and Zhuang adolescents (*p* < 0.01). Additionally, meeting any number of guidelines was significantly associated with enhanced PF indicators in rural adolescents (*p* < 0.001). Meeting all three guidelines was significantly associated with enhanced PF indicators in adolescents from any residence (*B* > 0.44, *p* < 0.05). Meeting only one guideline was not significantly associated with enhanced PF indicators in both suburban and urban adolescents (*p* > 0.05). Meeting two guidelines was not significantly associated with enhanced flexibility in urban adolescents (*p* > 0.05).

**Table 2:** Associations between the number of guidelines met and self-reported PF indicators by ethnicity and residence.

Number of guidelines met	Characteristics	General physical fitness		Cardiorespiratory fitness		Muscular strength		Speed/agility		Flexibility	
		*B*	*p*	*B*	*p*	*B*	*p*	*B*	*p*	*B*	*p*
1	Han	0.15	<0.01	0.18	<0.01	0.18	<0.01	0.20	<0.01	0.20	<0.001
	Zhuang	0.13	<0.001	0.15	<0.001	0.08	<0.01	0.11	<0.001	0.10	<0.01
2	Han	0.24	<0.001	0.26	<0.001	0.29	<0.001	0.23	<0.01	0.30	<0.001
	Zhuang	0.30	<0.001	0.29	<0.001	0.19	<0.001	0.25	<0.001	0.23	<0.001
3	Han	0.70	<0.001	0.72	<0.001	0.78	<0.001	0.87	<0.001	0.88	<0.001
	Zhuang	0.74	<0.001	0.66	<0.001	0.64	<0.001	0.72	<0.001	0.54	<0.001
1	Rural	0.14	<0.001	0.18	<0.001	0.12	<0.001	0.17	<0.001	0.15	<0.001
	Suburban	**0.13**	**0.103**	**0.12**	**0.150**	**0.07**	**0.699**	**0.07**	**0.747**	**0.10**	**0.370**
	Urban	**0.11**	**0.160**	**0.11**	**0.195**	**0.07**	**0.667**	**0.08**	**0.586**	**0.06**	**0.842**
2	Rural	0.27	<0.001	0.28	<0.001	0.20	<0.001	0.23	<0.001	0.26	<0.001
	Suburban	0.35	<0.001	0.36	<0.001	0.26	<0.001	0.37	<0.001	0.33	<0.001
	Urban	0.29	<0.001	0.21	<0.01	0.22	<0.01	0.18	<0.05	**0.10**	**0.564**
3	Rural	0.94	<0.001	0.80	<0.001	0.81	<0.001	0.87	<0.001	0.80	<0.001
	Suburban	0.53	<0.01	0.47	<0.05	0.54	<0.01	0.68	<0.001	0.52	<0.01
	Urban	0.54	<0.01	0.63	<0.001	0.56	<0.001	0.58	<0.01	0.44	<0.05

Non-significant *B* values are indicated by a red dashed border around the cell.

Table 3 presents the associations between specific combinations of guidelines met and self-reported PF indicators, compared to meeting none of the guidelines, stratified by ethnicity and residence. The data show that among Han adolescents, meeting the screen time guideline alone or in combination with MVPA or sleep guidelines showed no significant association with the majority of PF indicators (*p* > 0.05 for most comparisons). Meeting the MVPA guideline alone was not significantly associated with general PF or flexibility (*p* > 0.05). Among Zhuang adolescents, no significant (*p > 0.05*) correlation exists between meeting the screen time or “MVPA + screen” guidelines and the muscular strength. In all Han and Zhuang participants, all other combinations are significantly (*p* < 0.001) correlated with all better PF indicators. In addition, any specific combinations of guidelines met was significantly (*p* < 0.001) associated with enhanced PF indicators in rural adolescents, except for muscular strength, which showed no significant association with adherence to the screen time only or “MVPA + screen” guidelines. In urban adolescents, meeting “Screen only,” “Sleep only,” and “Screen + sleep” combinations of guidelines were not significantly (*p* > 0.05) associated with all PF indicators.

**Table 3:** Associations between the specific combinations of guidelines met and self-reported PF indicators by residence and ethnicity.

Specific combinations of guidelines met	Characteristics	General physical fitness		Cardiorespiratory fitness		Muscular strength		Speed/agility		Flexibility	
		*B*	*p*	*B*	*p*	*B*	*p*	*B*	*p*	*B*	*p*
MVPA only	Han	**0.37**	**0.070**	0.51	<0.01	0.56	<0.001	0.44	<0.05	**0.30**	**0.333**
	Zhuang	0.49	<0.001	0.53	<0.001	0.39	<0.001	0.52	<0.001	0.35	<0.001
Screen only	Han	**0.06**	**1.000**	**0.10**	**1.000**	**0.07**	**1.000**	**0.19**	**0.228**	0.24	<0.05
	Zhuang	0.13	<0.05	0.15	<0.01	**0.06**	**0.933**	0.14	<0.01	0.16	<0.01
Sleep only	Han	0.16	<0.05	0.17	<0.05	0.18	<0.01	0.18	<0.05	0.19	<0.05
	Zhuang	0.11	<0.01	0.13	<0.001	**0.07**	**0.104**	0.08	<0.05	0.07	0.118
MVPA + screen	Han	**0.62**	**0.377**	**0.58**	**0.414**	**0.39**	**1.000**	**0.23**	**1.000**	**0.51**	**0.968**
	Zhuang	0.72	<0.001	0.74	<0.001	0.38	<0.01	0.84	<0.001	0.51	<0.001
MVPA + sleep	Han	0.60	<0.001	0.52	<0.001	0.62	<0.001	0.48	<0.01	0.40	<0.05
	Zhuang	0.52	<0.001	0.52	<0.001	0.44	<0.001	0.56	<0.001	0.40	<0.001
Screen + sleep	Han	**0.15**	**0.377**	**0.19**	**0.069**	0.21	<0.05	**0.18**	**0.129**	0.27	<0.01
	Zhuang	0.20	<0.001	0.18	<0.001	0.10	<0.05	0.11	<0.05	0.15	<0.001
All	Han	0.70	<0.001	0.72	<0.001	0.78	<0.001	0.87	<0.001	0.88	<0.001
	Zhuang	0.74	<0.001	0.66	<0.001	0.64	<0.001	0.72	<0.001	0.54	<0.001
MVPA only	Rural	0.55	<0.001	0.63	<0.001	0.38	<0.01	0.46	<0.001	0.37	<0.01
	Suburban	**0.27**	**0.531**	0.40	<0.05	0.48	<0.01	0.45	<0.01	**0.20**	**1.000**
	Urban	0.49	<0.01	0.45	<0.01	0.41	<0.01	0.49	<0.01	**0.34**	**0.084**
Screen only	Rural	0.14	<0.05	0.18	<0.01	**0.10**	**0.162**	0.18	<0.01	0.19	<0.001
	Suburban	**0.12**	**1.000**	**0.18**	**0.535**	**−0.01**	**1.000**	**0.18**	**0.497**	0.27	<0.05
	Urban	**0.02**	**1.000**	**−0.01**	**1.000**	**−0.02**	**1.000**	**0.04**	**1.000**	**0.06**	**1.000**
Sleep only	Rural	0.13	<0.001	0.17	<0.001	0.12	<0.01	0.16	<0.001	0.14	<0.001
	Suburban	**0.11**	**0.723**	**0.08**	**1.000**	**0.05**	**1.000**	**0.00**	**1.000**	**0.04**	**1.000**
	Urban	**0.10**	**0.868**	**0.10**	**0.703**	**0.06**	**1.000**	**0.04**	**1.000**	**0.02**	**1.000**
MVPA + screen	Rural	0.74	<0.001	0.68	<0.001	**0.41**	**0.102**	0.61	<0.01	0.50	<0.05
	Suburban	**0.57**	**0.155**	0.82	<0.01	**0.25**	**1.000**	0.98	<0.001	0.85	<0.01
	Urban	0.74	<0.01	0.65	<0.01	**0.40**	**0.326**	0.71	<0.01	**0.22**	**1.000**
MVPA + sleep	Rural	0.60	<0.001	0.63	<0.001	0.50	<0.001	0.58	<0.001	0.56	<0.001
	Suburban	0.53	<0.001	0.56	<0.001	0.43	<0.01	0.53	<0.001	**0.34**	**0.055**
	Urban	0.41	<0.001	0.29	<0.05	0.41	<0.001	0.43	<0.001	**0.12**	**1.000**
Screen + sleep	Rural	0.17	<0.001	0.18	<0.001	0.12	<0.05	0.13	<0.05	0.19	<0.001
	Suburban	0.28	<0.01	0.26	<0.01	0.21	<0.05	0.26	<0.01	0.28	<0.01
	Urban	**0.17**	**0.243**	**0.11**	**1.000**	**0.10**	**1.000**	**−0.02**	**1.000**	**0.07**	**1.000**
All	Rural	0.94	<0.001	0.80	<0.001	0.81	<0.001	0.87	<0.001	0.80	<0.001
	Suburban	0.53	<0.05	0.47	<0.05	0.54	<0.01	0.68	<0.001	0.52	<0.05
	Urban	0.54	<0.01	0.63	<0.001	0.56	<0.01	0.58	<0.01	0.44	0.053

Non-significant *B* values are indicated by a red dashed border around the cell.

## 4 Discussion

This study aimed to investigate the associations between adherence to the 24-h movement guidelines and self-reported PF among Han and Zhuang ethnic adolescents in southern China, as well as to explore potential differences by residence and ethnicity. The findings of the current study are as follows: (i) A very small proportion of adolescents met the three 24-h movement guidelines of MVPA, screen time, and sleep duration. (ii) Greater adherence to the 24-h movement guidelines was significantly associated with higher levels of self-reported PF indicators, demonstrating a dose–response relationship (i.e., 3 > 2 > 1 > 0). (iii) Adherence to the MVPA, screen time, and sleep duration guidelines independently was significantly associated with better self-reported PF indicators. However, the effects of adherence to the screen time and sleep duration guidelines appeared to be obscured in the pairwise comparisons of the associations between the specific combinations of guidelines met and self-reported PF indicators. Furthermore, this association may primarily depend on meeting the MVPA guideline, followed by screen time and sleep duration. (iv) Ethnic differences had a minimal influence on the relationship between guidelines adherence and self-reported PF indicators, whereas residence differences significantly impacted the associations between adherence to the “Screen only,” “Sleep only,” and “Screen + Sleep” guidelines and self-reported PF indicators.

### 4.1 The prevalence of adhering to the 24-h movement guidelines

In this sample, only 1.6% of adolescents met the overall guidelines. This result was similar to the findings of two other studies on Chinese children and adolescents (29, 46), but significantly lower than those reported in studies from other countries (31, 32, 59, 60). A nationwide survey in China reported that 12.4% of adolescents aged 13–22 fully adhered to all guidelines, whereas adherence among those aged 13–17 was markedly lower (3.7%). This disparity may be associated with intense academic pressures in primary and secondary education settings and may also reflect younger adolescents’ limited self-regulatory capacity (30). Because the present data were collected exclusively in Guangxi, the even lower adherence observed here might additionally reflect the region’s relatively underdeveloped economy and lower health literacy (40).

In terms of meeting individual movement guidelines, 2.4, 10.1, and 37.9% of participants met the MVPA guideline, the screen time guideline, and the sleep duration guideline, respectively. Only the prevalence of meeting the sleep duration guideline was higher than the world average (17.7%), while the proportions of meeting the MVPA and screen time guidelines were both lower than the world average (7.7% for MVPA and 19.5% for screen time). More details are shown in Supplementary Table S3. These findings suggest that the low overall adherence is driven primarily by non-compliance with MVPA and screen-time recommendations.

Taken together, the results highlight a critical public health concern that warrants immediate attention and targeted interventions. Future efforts should therefore focus on enhancing guideline-adherence awareness among younger adolescents and developing programs specifically targeting MVPA and screen time behaviors.

### 4.2 Implications of adherence to the 24-h movement guidelines on self-reported PF indicators

The results of this study show that adhering to any number of the 24-h movement guidelines is more likely to lead to a higher level of PF, which suggests that sufficient physical activity, limited screen time, and appropriate sleep are related to the improvement of PF. However, previous studies (29, 31, 32) have indicated that meeting only one guideline, the screen time guideline, or the sleep duration guideline is not associated with PF improvement, which contrasts with the findings of our study. This discrepancy may be attributed to differences in experimental design or study population characteristics. Notably, although adherence to a single guideline was associated with higher PF, the effect size was small (*B* < 0.162), suggesting the association may be statistically detectable but of limited practical importance. Furthermore, this study reveals that meeting a greater number of the 24-h movement guidelines is increasingly and positively correlated with better PF indicators (i.e., 3 > 2 > 1 > 0). Meeting all three guidelines was associated with a PF gain of at least 0.44 points, exceeding the 0.405-point threshold for a moderate and meaningful improvement. These findings underscore the importance of adhering to all three 24-h movement guidelines (20) and provide empirical support for their holistic application in public health strategies.

In addition, all the combinations containing the MVPA guideline were significantly associated with PF indicators enhancement compared to those without the MVPA guideline (*B* > 0.22). This directly illustrates the leading role of meeting MVPA guideline in improving PF, which is consistent with the findings of Chen et al. (29), Cai et al. (30) and Tapia-Serrano et al. (31). Previous systematic reviews reported that there was strong, consistent evidence that MVPA was favorably associated with PF (20, 61). Specifically, MVPA enhances cardiorespiratory fitness via cardiorespiratory adaptation (62, 63), boosts muscular fitness (64, 65), and optimizes body composition (66, 67). These synergistic mechanisms collectively drive the significant association between MVPA and PF (20, 61). Moreover, increasing MVPA is likely to displace sedentary behaviors (including screen time) and improve sleep, thereby generating a synergistic effect that further enhances PF (30, 47, 68).

Interestingly, adherence to either the screen time or sleep duration guideline alone was significantly associated with improved PF. However, these associations tended to become non-significant or attenuated in pairwise comparisons of specific guideline combinations—a phenomenon that may reflect statistical interactions or mutual adjustment when other behaviors (e.g., MVPA) are concurrently considered. Notably, the associations with screen time adherence appeared more consistent across PF indicators than those with sleep duration adherence. Yet, the effect sizes for both guidelines were small (*B* < 0.18), suggesting limited practical relevance. Currently, controversies surround the relationships between screen time, sleep duration, and PF, respectively (22, 29, 30, 32). Some studies have reported a negative correlation between screen time and PF (45, 69, 70), whereas others have found a positive correlation between sleep duration and specific PF indicators (e.g., cardiorespiratory fitness, muscular fitness, and flexibility) (71). In detail, screen time (a major sedentary behavior) reduces the duration of MVPA, which may in turn impair PF (66). In contrast, sleep enhances PF by promoting nutrient absorption, regulating hormonal balance, modulating neural activity, and facilitating fatigue recovery—benefits that are especially prominent in children (20, 28).

Collectively, the data from the present study suggest that the effects of adhering to the 24-h movement guidelines on PF primarily depend on MVPA, followed by screen time and sleep duration. However, this is the first study of its kind to conduct pairwise comparisons among different combinations of meeting the 24-h movement guidelines. It is expected that more studies will be carried out in the future to clarify the relationship between meeting the 24-h movement guidelines and PF.

### 4.3 Ethnic and residential differences in the association between adherence to the 24-h movement guidelines and self-reported PF indicators

Minimal differences in the association between the 24-h movement guidelines and PF were observed across ethnic groups. This may be because all participants lived in the same geographic region. Despite differing ethnic backgrounds, shared environmental factors likely led to similar lifestyle patterns.

However, adherence to the screen time, “MVPA + screen” or “Screen + sleep” guidelines was not associated with improvements in most PF indicators among Han adolescents, a pattern not seen in Zhuang adolescents. Several explanations may account for this disparity. First, this may indicate differences in screen behavior patterns between the two groups. Among parents of Han adolescents in China, expectations for their children’s academic achievement are relatively higher (72, 73), and this parental expectation may contribute to increased screen-based learning behaviors and longer weekend screen time among Han adolescents (20, 74). Such differences could weaken the beneficial effects of adhering to screen guidelines on PF, primarily by elevating adolescents’ academic pressure and reducing their engagement in PA (20, 75). Second, given the relatively small effect size (*B* < 0.19) of screen behaviors on PF, this association is highly susceptible to interference from other confounding factors. For instance, although both groups reside in the same broader region, Zhuang adolescents were more likely to live in rural areas and experience lower socioeconomic conditions (42). In these settings, lower parental education and limited household resources may restrict digital access and structured academic activities (72, 73), while traditional customs, such as participation in festivals, folk singing, and communal dancing, are more likely to be preserved (43). These communities also tend to maintain a distinct dietary culture (41, 42). In contrast, Han adolescents may face higher academic demands and calorie intake due to urban residence and higher socioeconomic status (76, 77), which could indirectly suppress PF despite guideline adherence. While these socioeconomic gradients were not measured in our study, they may underlie the observed differences in the association between movement behaviors and PF, suggesting that the findings reflect not just cultural variation, but also social context.

In summary, although unmeasured socioeconomic factors may contribute to contextual differences, our findings indicate that adherence to screen time, “MVPA + screen” or “Screen + sleep” guidelines does not confer the same PF benefits among Han adolescents as observed in their Zhuang peers. This disparity likely reflects differences in behavioral context, such as socioeconomic status, parental education, family structure, academic pressure, and cultural practices, and highlights the need for integrated, context-sensitive approaches that move beyond isolated movement behavior recommendations.

Another finding of this study is that significant differences exist in the relationship between meeting the 24-h movement guidelines and multiple PF indicators across residence groups. Among rural adolescents, adherence to any combination of guidelines was significantly associated with higher PF level. In contrast, among urban adolescents, no significant association was observed between adherence to screen time guideline, sleep guideline, or their combination and improvements in the majority of PF indicators. First, a large body of research has demonstrated the existence of urban–rural disparities in adolescents’ screen behaviors and sleep quality (71, 74). Urban adolescents often face higher academic expectations, which may contribute to increased screen-based learning activities, sedentary behaviors, and poorer sleep quality (71, 78, 79). Therefore, it is reasonable to hypothesize that the beneficial effects of adhering to screen time and sleep guidelines on PF may be weakened among urban adolescents—a phenomenon potentially driven by their elevated academic stress and reduced levels of PA (66, 71). Furthermore, urban adolescents in China generally have higher socioeconomic status than their rural peers, which contributes to more diverse lifestyles and potentially greater exposure to PF-related risk factors (34). For instance, urban adolescents tend to have a higher intake of calorie-dense foods (76), engage in less active commuting (e.g., walking or cycling) (34), and experience greater academic pressure (33, 51). Collectively, the influence of these factors may weaken the association between adherence to screen time and sleep guidelines and PF among urban adolescents.

However, due to the lack of data on these potential confounders, we are unable to investigate the underlying mechanisms. Therefore, future research is needed to examine how screen behavior patterns and sleep quality relate to PF in this population. It should also explore how these associations vary by socioeconomic status, with the aim of informing equitable, evidence-based interventions aligned with the 24-h movement guidelines. Furthermore, although the associations of adherence to screen time, sleep duration, and their combination with PF differed significantly between urban and rural areas, the effect size differences were relatively small (*B* < 0.19), suggesting limited practical significance.

### 4.4 Limitations and strengths

This study has some inherent limitations. First, the cross-sectional research design cannot infer causal relationships between variables. Longitudinal tracking or experimental studies are required to determine the directionality of the association between meeting the 24-h movement guidelines and PF. Second, self-reported data may be affected by subjects’ recall bias and social desirability. Objectively measured devices can avoid such biases. Third, the use of convenience sampling may limit the representativeness of the sample, as it may not fully reflect the characteristics of Han and Zhuang adolescents. Future studies should employ more rigorous sampling methods (e.g., stratified random sampling) and larger sample sizes to enhance the generalizability of the findings. Finally, some important confounding factors (such as dietary habits, socioeconomic status, parental education, peer support, family structure and built environment) were not included in the investigation, which may limit the interpretation of the research results. Nevertheless, this study also has several strengths that need to be emphasized. First, it is the first large-sample exploration to focus on Han and Zhuang adolescents, expanding the scope of the population for understanding adherence to the 24-h movement guidelines. Second, it is the first pairwise comparison of the number and combination of adhering to the 24-h movement guideline. The relationships between different numbers and combinations regarding the 24-h movement guidelines and PF are more intuitive and reliable. Third, it is the first exploration of the impact of ethnicity and place of residence on the relationship between adhering to the 24-h movement guidelines and PF, providing more valuable clues for the promotion and optimization of the 24-h movement guidelines.

## 5 Conclusion

Meeting more of the recommendations in the 24-h movement guidelines is incrementally and positively correlated with better self-reported PF indicators in adolescents of Chinese Han and Zhuang ethnicities. This positive association is primarily determined by MVPA, followed by screen time and sleep duration. In addition, the associations between adhering to the 24-h movement guidelines and self-reported PF are similar between the Han and Zhuang ethnic groups, but there are differences between urban, suburban, and rural regions. Given that only 1.6% of the sample meets all three guidelines, it is urgently necessary to actively promote the implementation of these guidelines.

## Data Availability

The original contributions presented in the study are included in the article/Supplementary material, further inquiries can be directed to the corresponding author.
