# Supplementary material for: Ethnic and residential differences in adherence to the 24-h movement guidelines and self-reported physical fitness among Zhuang and Han adolescents in southern China
Source: Front Public Health. 2025 Dec 4;13:1631277. doi: 10.3389/fpubh.2025.1631277 (PMC12711711; doi:10.3389/fpubh.2025.1631277)
Supplement: Supplementary file 1 [file Data_Sheet_1.docx]

**Ethnic and Residential Differences in Adherence to the 24-hour Movement Guidelines and Self-Reported Physical Fitness among Zhuang and Han Adolescents in Southern China**

**TABLE S1** Descriptive statistics and thresholds for meaningful differences in self-reported PF items

| **PF Items** | **M****±SD** | **Commonly Used Threshold (0.5 × SD) ^#^** | **Sensitive Threshold (0.2 × SD) ^$^** |
| --- | --- | --- | --- |
| General physical fitness | 3.53±0.809 | 0.405 | 0.162 |
| Cardiorespiratory fitness | 3.46±0.813 | 0.407 | 0.163 |
| Muscular strength | 3.32±0.780 | 0.390 | 0.156 |
| Speed/agility | 3.39±0.833 | 0.417 | 0.167 |
| Flexibility | 3.22±0.812 | 0.406 | 0.162 |
| **Overall PF (mean)** | **3.38±0.810** | **0.405** | **0.162** |

Note: Thresholds are derived from the observed standard deviation (SD) for each item on a 5-point Likert scale (1 = very poor, 5 = excellent). # Commonly used threshold: 0.5 SD, reflecting a moderate effect size considered meaningful in public health research. $ Sensitive threshold: 0.2 SD, representing a small but detectable change, particularly relevant in population-level analyses.

**TABLE S2** Distribution of adherence to the 24-hour movement guidelines and self-reported PF across ethnic groups and residential areas

| Variables | | Ethnicity | | Statistical value | *p* value | Residence | | | Statistical value | *p* value |
| --- | --- | --- | --- | --- | --- | --- | --- | --- | --- | --- |
|  |  | Han | Zhuang |  |  | Rural | Suburban | Urban |  |  |
| Adherence to the 24-hour movement guidelines | None | 411(27.6) | 1526(28.1) | χ2=0.136 | 0.713 | 1187(27.2) | 362(29.1) | 388(29.6) | χ2=3.526 | 0.172 |
|  | One | 738(49.6) | 2744(50.6) | χ2=0.414 | 0.520 | 2276(52.2) | 596(47.9) | 610(46.5) | χ2=17.205 | ＜0.001 |
|  | Two | 301(20.2) | 1080(19.9) | χ2=0.083 | 0.773 | 842(19.3) | 258(20.7) | 281(21.4) | χ2=3.261 | 0.196 |
|  | Three | 37(2.5) | 76(1.4) | χ2=8.586 | ＜0.01 | 51(1.2) | 28(2.3) | 34(2.6) | χ2=16.211 | ＜0.001 |
|  | MVPA only | 44(3.0) | 125(2.3) | χ2=2.101 | 0.147 | 66(1.5) | 49(3.9) | 54(4.1) | χ2=42.746 | ＜0.001 |
|  | Screen only | 166(11.2) | 529(9.7) | χ2=2.581 | 0.108 | 442(10.1) | 106(8.5) | 147(11.2) | χ2=5.168 | 0.075 |
|  | Sleep only | 528(35.5) | 2090(38.5) | χ2=4.496 | ＜0.05 | 1768(40.6) | 441(35.5) | 409(31.2) | χ2=41.970 | ＜0.001 |
|  | MVPA + screen | 9(0.6) | 53(1.0) | χ2=1.813 | 0.178 | 27(0.6) | 15(1.2) | 20(1.5) | χ2=10.893 | ＜0.01 |
|  | MVPA + sleep | 52(3.5) | 253(4.7) | χ2=3.761 | 0.052 | 153(3.5) | 59(4.7) | 93(7.1) | χ2=30.893 | ＜0.001 |
|  | Screen + sleep | 240(16.1) | 774(14.3) | χ2=3.279 | 0.070 | 662(15.2) | 184(14.8) | 168(12.8) | χ2=4.670 | 0.097 |
| General physical fitness | Very poor | 28(1.9) | 65(1.2) | χ2=4.938 | 0.294 | 61(1.4) | 18(1.4) | 14(1.1) | χ2=22.108 | ＜0.01 |
|  | Poor | 41(2.8) | 173(3.2) |  |  | 128(2.9) | 42(3.4) | 44(3.4) |  |  |
|  | Average | 737(49.6) | 2727(50.3) |  |  | 2223(51) | 612(49.2) | 629(47.9) |  |  |
|  | Good | 485(32.6) | 1750(32.3) |  |  | 1399(32.1) | 428(34.4) | 408(31.1) |  |  |
|  | Very good | 196(13.2) | 711(13.1) |  |  | 545(12.5) | 144(11.6) | 218(16.6) |  |  |
| Cardiorespiratory fitness | Very poor | 23(1.5) | 70(1.3) | χ2=2.024 | 0.731 | 68(1.6) | 12(1.0) | 13(1.0) | χ2=19.280 | ＜0.05 |
|  | Poor | 77(5.2) | 292(5.4) |  |  | 217(5) | 77(6.2) | 75(5.7) |  |  |
|  | Average | 741(49.8) | 2772(51.1) |  |  | 2263(52) | 632(50.8) | 618(47.1) |  |  |
|  | Good | 483(32.5) | 1679(30.9) |  |  | 1345(30.9) | 373(30) | 444(33.8) |  |  |
|  | Very good | 163(11.0) | 613(11.3) |  |  | 463(10.6) | 150(12.1) | 163(12.4) |  |  |
| Muscular strength | Very poor | 29(2.0) | 83(1.5) | χ2=5.890 | 0.208 | 75(1.7) | 17(1.4) | 20(1.5) | χ2=26.064 | ＜0.01 |
|  | Poor | 103(6.9) | 397(7.3) |  |  | 310(7.1) | 109(8.8) | 81(6.2) |  |  |
|  | Average | 810(54.5) | 3088(56.9) |  |  | 2521(57.9) | 672(54) | 705(53.7) |  |  |
|  | Good | 437(29.4) | 1450(26.7) |  |  | 1148(26.4) | 359(28.9) | 380(28.9) |  |  |
|  | Very good | 108(7.3) | 408(7.5) |  |  | 302(6.9) | 87(7.0) | 127(9.7) |  |  |
| Speed/Agility | Very poor | 25(1.7) | 80(1.5) | χ2=4.729 | 0.316 | 72(1.7) | 13(1.0) | 20(1.5) | χ2=41.285 | ＜0.001 |
|  | Poor | 103(6.9) | 438(8.1) |  |  | 362(8.3) | 90(7.2) | 89(6.8) |  |  |
|  | Average | 781(52.5) | 2784(51.3) |  |  | 2280(52.3) | 661(53.1) | 624(47.5) |  |  |
|  | Good | 437(29.4) | 1542(28.4) |  |  | 1250(28.7) | 335(26.9) | 394(30.0) |  |  |
|  | Very good | 141(9.5) | 582(10.7) |  |  | 392(9) | 145(11.7) | 186(14.2) |  |  |
| Flexibility | Very poor | 33(2.2) | 128(2.4) | χ2=2.151 | 0.708 | 98(2.2) | 30(2.4) | 33(2.5) | χ2=22.510 | ＜0.01 |
|  | Poor | 163(11.0) | 551(10.2) |  |  | 457(10.5) | 131(10.5) | 126(9.6) |  |  |
|  | Average | 834(56.1) | 3143(57.9) |  |  | 2565(58.9) | 712(57.2) | 700(53.3) |  |  |
|  | Good | 351(23.6) | 1221(22.5) |  |  | 949(21.8) | 289(23.2) | 334(25.4) |  |  |
|  | Very good | 106(7.1) | 383(7.1) |  |  | 287(6.6) | 82(6.6) | 120(9.1) |  |  |

**TABLE S3** Proportion of participants meeting the 24-hour movement guidelines by country (%)

| **Country** | **Adherence to the 24-hour movement guidelines** | | | | | | | | | |
| --- | --- | --- | --- | --- | --- | --- | --- | --- | --- | --- |
|  | **Number of guidelines** | | | | **Specific combination of guidelines** | | | | | |
|  | **0** | **1** | **2** | **3** | **MVPA only** | **Screen only** | **Sleep only** | **MVPA + screen** | **MVPA + sleep** | **Screen + sleep** |
| Canada(1) | 11.0 | 34.9 | 37.0 | 17.1 | 5.2 | 5.4 | 24.3 | 2.9 | 11.3 | 22.8 |
| Spain(2) | 9.9 | 49.9 | 34.5 | 5.7 | 4.9 | 1.7 | 43.3 | 0.9 | 26.5 | 7.1 |
| China(3) | 36.0 | 48.8 | 14.3 | 0.9 | 1.8 | 29.8 | 17.2 | 1.9 | 1.9 | 10.5 |
| China_1(4) | 28.7 | 54.6 | 15.1 | 1.7 | 2.6 | 45.1 | 6.9 | 4.1 | 0.9 | 10.1 |
| China_2(5) | 9.9 | 44.4 | 33.4 | 12.4 | 4.3 | 30.3 | 9.7 | 8.0 | 6.3 | 19.0 |
| Czech(6) | 33.0 | 37.7 | 22.8 | 6.5 | 18.3 | 11.0 | 8.4 | 8.2 | 9.3 | 5.3 |
| Global(7) | 19.0 | 43.9 | 29.9 | 7.2 | 16.5 | 13.2 | 14.2 | 9.4 | 11.0 | 9.5 |
| Average | 21.1 | 44.9 | 26.7 | 7.4 | 7.7 | 19.5 | 17.7 | 5.1 | 9.6 | 12.0 |
| **Present study** | **28.0** | **50.4** | **20.0** | **1.6** | **2.4** | **10.1** | **37.9** | **0.9** | **4.4** | **14.7** |

# Reference

1. Jakubec L, Gába A, Dygrýn J, Rubín L, Šimůnek A, Sigmund E. Is Adherence to the 24-Hour Movement Guidelines Associated with a Reduced Risk of Adiposity among Children and Adolescents? *BMC public health* (2020) 20(1):1119. doi: 10.1186/s12889-020-09213-3.

2. Tapia-Serrano M, López-Gil JF, Sevil-Serrano J, García-Hermoso A, Sánchez-Miguel PA. What Is the Role of Adherence to 24-Hour Movement Guidelines in Relation to Physical Fitness Components among Adolescents? *Scand J Med Sci Sports* (2023) 33(8):1373-83. doi: 10.1111/sms.14357.

3. Chen Z, Chi G, Wang L, Chen S, Yan J, Li S. The Combinations of Physical Activity, Screen Time, and Sleep, and Their Associations with Self-Reported Physical Fitness in Children and Adolescents. *Int J Environ Res Public Health* (2022) 19(10). doi: 10.3390/ijerph19105783.

4. Chen S, Liang K, López-Gil JF, Drenowatz C, Tremblay MS. Association between Meeting 24-H Movement Guidelines and Academic Performance in a Sample of 67,281 Chinese Children and Adolescents. (2024) 24(4):487-98. doi: 10.1002/ejsc.12034.

5. Cai S, Zhong P, Dang J, Liu Y, Shi D, Chen Z, et al. Associations between Combinations of 24-H Movement Behaviors and Physical Fitness among Chinese Adolescents: Sex and Age Disparities. *Scandinavian Journal of Medicine & Science in Sports* (2023) 33(9):1779-91. doi: 10.1111/sms.14427.

6. Tanaka C, Tremblay MS, Okuda M, Tanaka S. Association between 24-Hour Movement Guidelines and Physical Fitness in Children. *Pediatr Int* (2020) 62(12):1381-7. doi: 10.1111/ped.14322.

7. Roman-Viñas B, Chaput JP, Katzmarzyk PT, Fogelholm M, Lambert EV, Maher C, et al. Proportion of Children Meeting Recommendations for 24-Hour Movement Guidelines and Associations with Adiposity in a 12-Country Study. *The international journal of behavioral nutrition and physical activity* (2016) 13(1):123. doi: 10.1186/s12966-016-0449-8.
